# The combinations of multiple factors to improve the diagnostic sensitivity and specificity after artificial joint infection

**DOI:** 10.1186/s13018-020-01669-8

**Published:** 2020-04-25

**Authors:** Lei Chu, You-Liang Ren, Jun-Song Yang, Jin Yang, Hang Zhou, Hai-Tao Jiang, Lei Shi, Ding-Jun Hao, Zhong-Liang Deng

**Affiliations:** 1 Department of Orthopedic Surgery, Second Affiliated Hospital, Chongqing Medical University, Chongqing, 40010 China; 2 Department of spinal surgery, Honghui hospital, Xi’an Jiaotong University, No.76, Nanguo Road, Beilin District, Xi’an, 710054 Shaanxi China

**Keywords:** Diagnosis, Regional characteristics, Methicillin-resistant *Staphylococcus aureus* (MRSA), Antibiotic resistance, After artificial joint

## Abstract

**Objective:**

To discuss the sensitivity and specificity of the combinations of multiple factors that work on bone infection after artificial joint, and provide evidence-based medical basis for the early diagnosis of infection after artificial joint.

**Methods:**

A retrospective review was conducted on 35 patients diagnosed with periprosthetic joint infections (PJI) or aseptic loosening (AL) who both received revision operation from January 2011 to January 2015. Analyzing and comparing their epidemiology indexes and expounded a series of auxiliary examinations corresponding positive diagnosis ratio.

**Results:**

Thirty-five patients were divided into two groups. One is called group PJI which includes 16 patients, and the other is called group AL which contains 19 patients. There was no statistical difference between in age (*p* = 0.536), gender ratio (*p* = 0.094), and the time of catching infection or getting loose (*p* = 0.055). Swelling was statistical significant (*p* = 0.0435 < 0.05). AUC of CRP = 0.947, ESR = 0.893, IL-6 = 0.893, PCT = 0.781, WBC = 0.839, and PMN = 0.755, respectively, CRP has a high diagnostic value to PJI, ESR, IL-6, PCT, WBC, and PMN% possess a moderate diagnostic value. There were 3 cases of PJI whose pathological paraffin section showed infectious inflammatory cells (100%). three PJI patients and one AL patient whose 99mTc-MDP examination presented 100% infection or looseness rate.

**Conclusion:**

CRP has a high diagnostic value to PJI. Histopathology HE staining, Gram staining, and 99mTc-MDP provide a highly accurate diagnosis for PJI. Therefore, the results suggest combining the unique clinical symptoms of PJI patients with relevant laboratory indexes, histopathologic characteristics, and imageological examinations that can improve diagnostic sensitivity and specificity of PJI in its early stage.

## Introduction

Periprosthetic joint infection (PJI) is one of the most devastating and costly complications following total knee arthroplasty (TKA) or total hip arthroplasty (THA), which is still commonly regarded as a challenge, especially in terms of diagnosis. Facing the challenge of accurately diagnosing PJI, the Musculoskeletal Infection Society (MSIS) published a definition of PJI in 2011. Diagnosis of PJI is multifactorial and relies on many results. Currently, surgeons utilize a wide spectrum of methods in an attempt to diagnose PJI, including history and clinical features, local measures of synovial inflammation (synovial fluid white blood cell (WBC) count and differential, synovial tissue histology), serologic tests of inflammation indicators like WBC count and polymorphonuclear (PMN) portion, C-reactive protein (CRP) level, erythrocyte sedimentation rate (ESR), interleukin-6 (IL-6) and procalcitonin (PCT), radiographic tests (radiographs, bone scan, magnetic resonance imaging (MRI), computed tomography (CT), positron emission tomography) and ultrasound scan, and bacterial isolation techniques (intra-operative culture) [1–5]. While all above current methods for diagnosis of PJI may lack sensitivity and specificity, and the limited sensitivity and specificity of the available tests make it difficult to distinguish between PJI and other causes of prosthetic failure, such as metal allergy or aseptic loosening (AL), so there is still no “gold standard for diagnosis of PJI [2], there are several reasons for this, including the absence of specific clinical signs and symptoms, the relative lack of accurate laboratory tests, and difficulties in culture isolation of pathogens due to prior therapy and formation of biofilms, particularly in low-grade infections that often cause false-negative results [3].

Our study is the first retrospective multiple factors study to explore the diagnostic value of different tests in the southwest of China. The aim is to provide evidence-based medical basis for the early diagnosis of infection after artificial joint.

## Material and methods

All the researches about patients’ information were approved by the Ethics Committee of our hospital. We systematically analyzed 35 patients who underwent revision arthroplasty surgery for PJI or aseptic loosening after total hip and knee arthroplasty from January 2010 until January 2015. There were 14 men and 21 women. According to the standardized definition of PJI, dividing 35 patients into two groups, the PJI group and the AL group, and combining with the results of culture, histology, laboratory tests, radiographic tests, ultrasound, and so on.

The detail of demographics, like age, sex, the proportion of hip and knee cases in every group, the amount of days before they occurred PJI or AL, and the physical examination were recorded in both two groups.

The laboratory tests, including the WBC count, differential (PMN%), ESR, CRP, IL-6, and PCT were also recorded to analyze the sensitivity (SN), specificity (SP), positive predictive value (PPV), and negative predictive value (NPV). Receiver operating characteristic curves were used to examine the relationship between sensitivity and the false-positive rate (1-specificity), and analyzing the sensitivity and specificity of different combinations of all six kinds of tests, and asking whether the different combinations had similar sensitivity, specificity to diagnosis PJI and whether there were statistic differences of between different combinations and single test.

All specimens were analyzed using standard microbiological cultures. Cultures must be obtained from various tissues such as synovium, synovial fluid, intramedullary tissue, granular tissue, and bone [6]. The culture was scored as positive if the same bacterial organism was identified in at least 3 different tissue or joint fluid samples [7].

The joint aspiration, intraoperative pathologic tissue with HE staining and Gram staining, radiographic examination and ultrasonography, and technetium-99m labeled methylene diphosphonate (99mTc-MDP) were also performed.

For one sign of PJI that includes joint effusions and fluid collections surrounding the hardware, ultrasonography has the ability to evaluate for periprosthetic collections as well as evaluate the regional relationships between the soft-tissue structures and implantable hardware [8]. So, ultrasonography could be measured, especially when suspected to periprosthetic collections. Power Doppler was used in this study, and it was used to detect the presence of these soft-tissue fluid collections, when they appear as a relatively thick hypoechoic band around the prosthesis on routine gray-scale imaging.

## Results

### History and clinical features

**Fig. 1:**
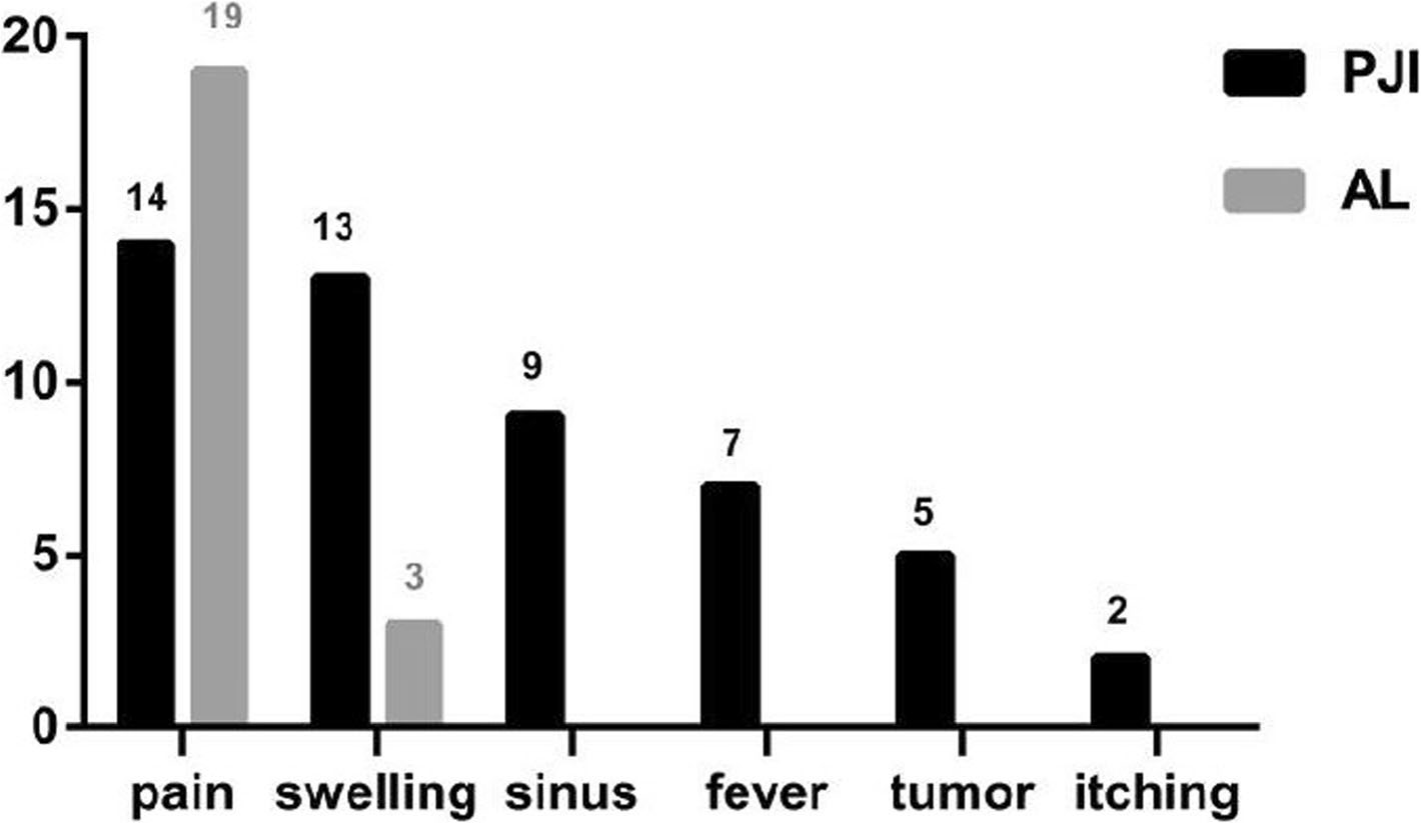
There was statistical difference in the clinical symptoms and distribution of two groups

We divided 35 patients into two groups, the one was the PJI group, included 16 patients, there were 9 males (44% and 7 females (56%), with a mean age of 66 years (range, 45–77 years), and the proportion of PJI after THA in this group was 56% (9 cases), and the other 7cases (44%) were after TKA. In the AL group, there were 5 males (26%) and 14 females (74%), and the mean age of this group was 64 years (range, 38–82 years) and it included 18 cases (95%) that had underwent THA, and the other one was TKA. There was no statistical difference in age (*p* = 0.536), sex (*p* = 0.094), occurring time after surgery (*p* = 0.055), and with significant difference in the proportion of the hip: knee between two groups (*p* = 0.010 < 0.05) (Table 1). For the clinical symptoms of two groups, there were apparently distinction and with significant difference (*p* = 0.007 < 0.05). In the PJI group, the clinical symptoms included pain (14 cases, 88%), swelling (13 cases, 81%), sinus tract (9 cases, 56%), fever (7 cases, 44%), tumor (5 cases, 31%), and itching (2 cases, 12%). While in the AL group, there were only two kinds of symptoms, pain and swelling, and all AL cases owned the symptom of pain, and only 3 patients felt swelling (16%). For the clinical symptoms of two groups, there was statistical differences in swelling (*p* = 0.0435 < 0.05), with no significant difference in pain between two groups (*p* = 4.887) (Fig. 1).

**Table 1:**
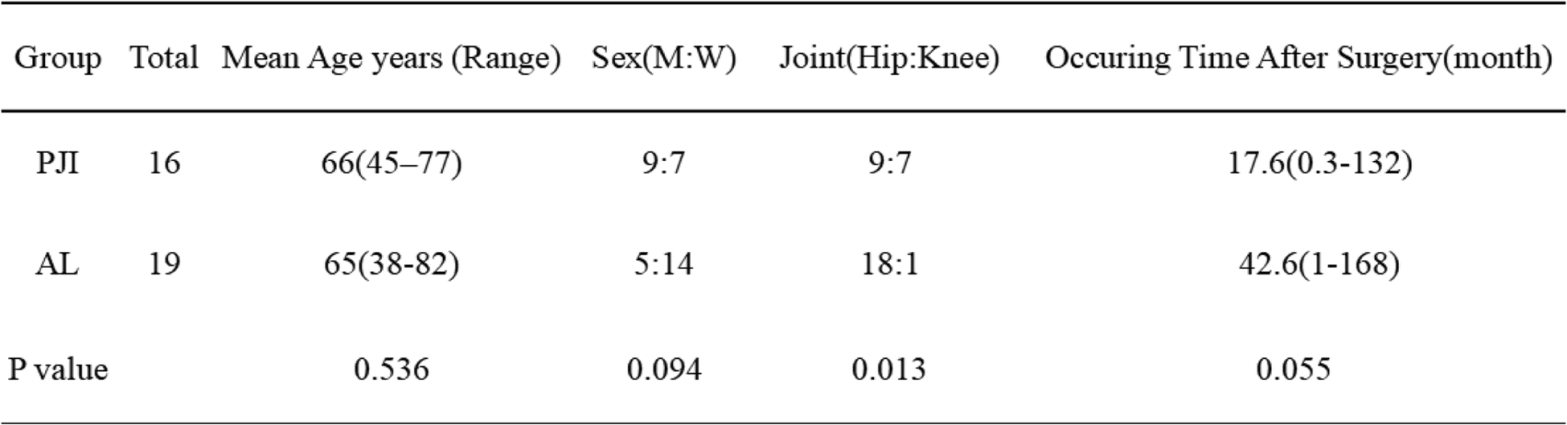
Demographics of patients

### Microbiological culture

In all 16 PJI patients, there were only 8 cases that had positive culture results, the positive value of bacteria culture was 50%, and there were 6 cases (75%) belonged to Gram-positive bacteria. The samples mainly came from wound secretion 6 cases (75%), two samples came from aspiration fluid (25%), and there were 9 patients accompanied with sinus tract during preoperative, so the positive value of bacteria culture of wound secretion was 67%. There were 7 PJI cases who had underwent aspiration, while there were only two culture results came from aspiration, so the rate of positive was 12%. The other sample was intraoperative tissue, and the rate of positive was 6% (Table 2).

**Table 2:**
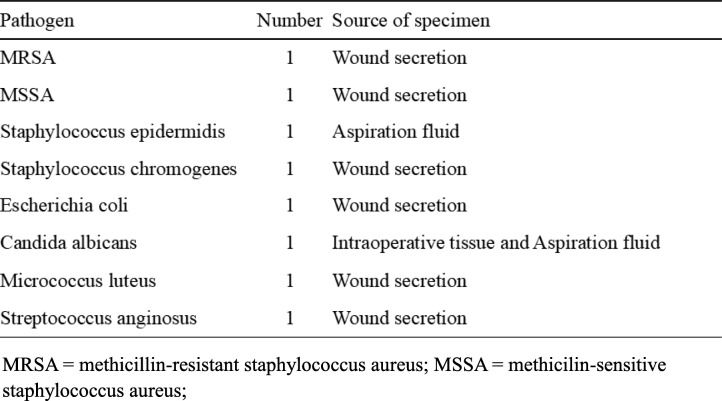
Culture results

*MRSA* methicillin-resistant *Staphylococcus aureus*, *MSSA* methicilin-sensitive *Staphylococcus aureus*

### Serologic laboratory tests

All patients of two groups had measured all serologic tests of inflammation indicators like WBC, PMN%, ESR, CRP, IL-6, and PCT, and analyzed the SN, SP, PPV, and NPV of all above tests. According to the results of the analysis, WBC and PMN% with the sensitivity of 19% and 38%, specificity of 100% and 95%, PPV of 100% and 86%, NPV of 59% and 64%, respectively. The sensitivity and specificity of ESR were 81% and 78%, and PPV (76%) and NPV (83%). The SN, SP, PPV, and NPV of preoperative serum CRP were 81%, 90%, 87%, and 85%. The IL-6 and PCT were 80% and 46%, 95% and 95%, 92% and 88%, and 86% and 69%, respectively. The highest sensitivity of tests was ESR and CRP, the value was 81%, the highest value of specificity and PPV were all 100%, all these were WBC, whereas the lowest value of sensitivity belonged to WBC as well (19%), and the highest value of NPV was IL-6 (86%) (Table 3).

**Table 3:**
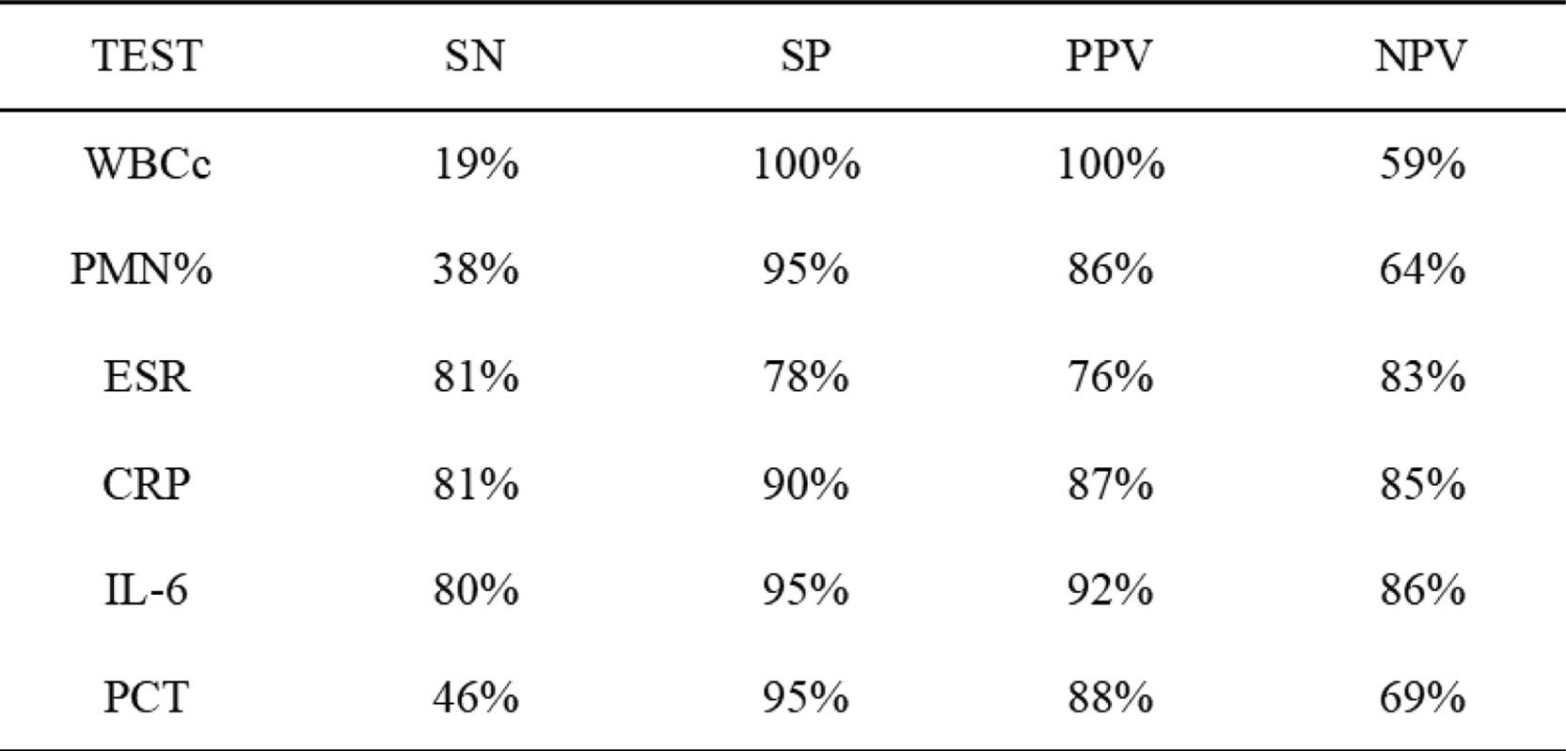
The SN, SP, PPV, and NPV of different tests

**Fig. 2:**
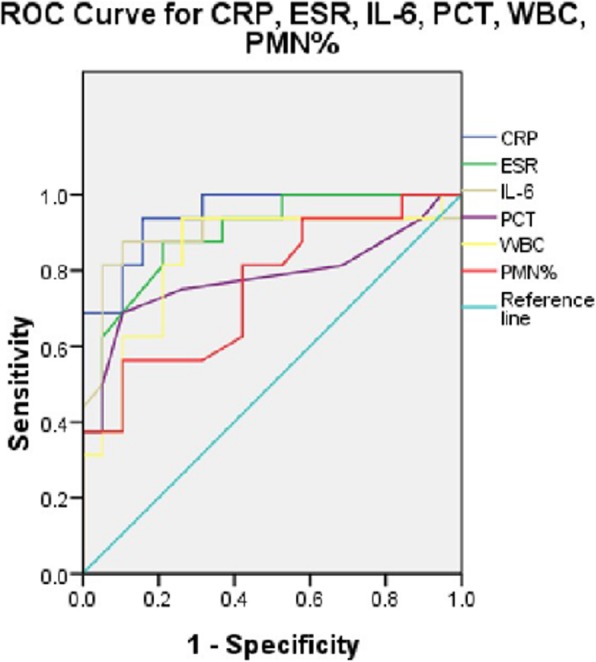
Receiver operating curves (ROC) of CRP, ESR, IL-6, PCT, WBC, and PMN%

Receiver operating characteristic (ROC) curves of different tests were plotted to examine the relationship between sensitivity and false-positive rate (100-specificity) based on the attributes of assignment into the PJI or AL group. The area under the curve (AUC) was calculated for assessing diagnosis utility. An AUC of 1 demonstrates an ideal test with 100% sensitivity and 100% specificity; an AUC of 0.9–1 indicates that this test yield to a high-quality diagnostic value; an AUC of 0.7–0.9 demonstrates that this test yield to a medium-quality diagnostic value and 0.5–0.7 belongs to low-quality diagnostic value, whereas an AUC of < 0.5 indicates that the diagnostic test is less useful: AUC of CRP = 0.947, AUC of ESR = 0.893, AUC of IL-6 = 0.893, AUC 0f PCT = 0.781, AUC of WBC = 0.839, AUC of PMN = 0.755. According to the AUC, CRP level yields to a high-quality diagnostic value, and ESR, IL-6, PCT, and WBC, and PMN% yield to a medium-quality diagnostic value (Fig. 2).

**Fig. 3:**
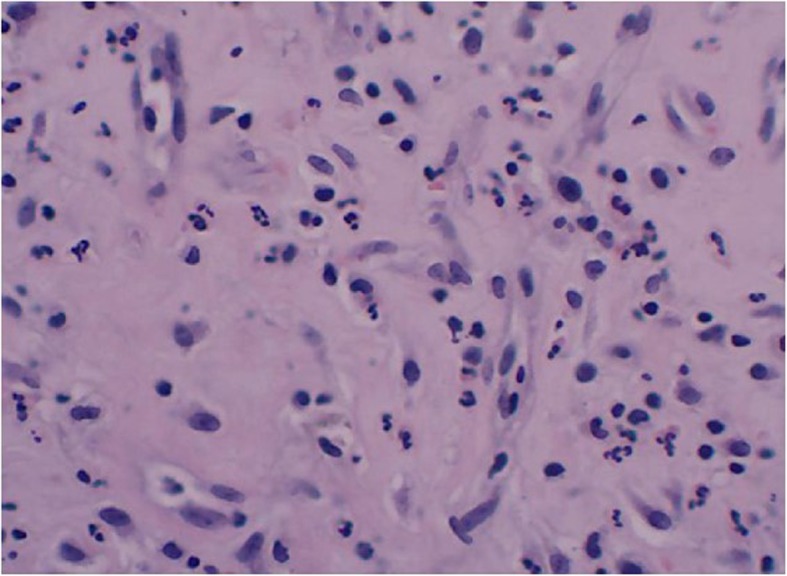
HE staining of PJI after THA

**Fig. 4:**
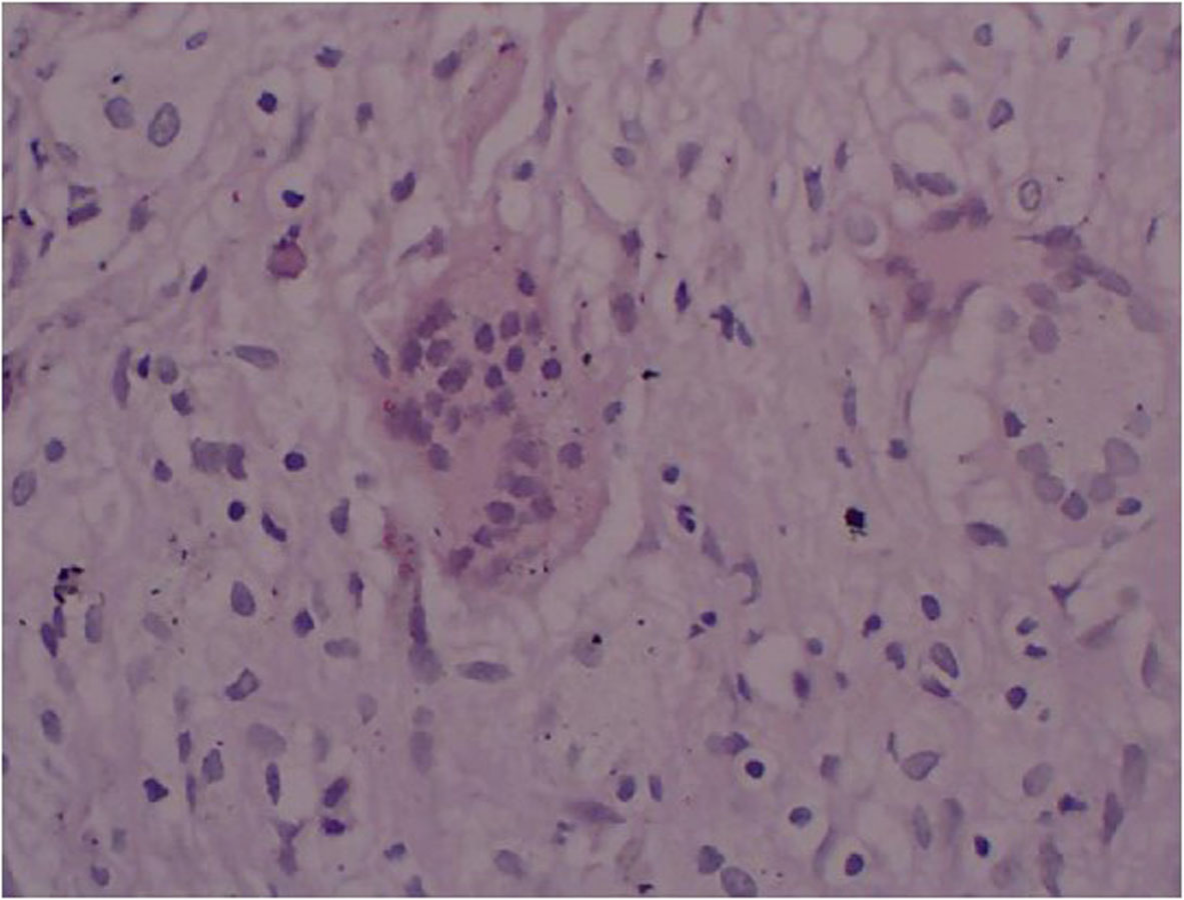
HE staining of AL after THA

By analyzing the different combinations of all six kinds of tests, we calculated the sensitivity and specificity of different combinations, and we found that the combination of PMN%, CRP, and IL-6 had the highest sensitivity (79%), while the highest value of specificity was 100%, and this result was the same as to the combinations of WBC + PMN%, WBC + PCT, PMN% + PCT, and WBC + PMN% + PCT (Table 4). According to the numbers of tests that combined, We divided all kinds of combinations into six groups, for example, the group of two tests indicates that this group includes all the combinations of any two kinds of tests. By comparing the sensitivity and specificity of six groups to each other, we found that there were no significant difference in sensitivity between the group of five tests and six tests, and this result indicates that there were no significant difference in sensitivity when we combined any five kinds of tests or more to together to diagnosis PJI, and this phenomenon did not exist in specificity (Table 5) (Figs. 3 and 4).

**Table 4:** Results of the different combinations of all tests

TEST	SN	SP
WBC + PMN%	25%	100%
WBC + ESR	25%	79%
WBC + CRP	25%	89%
WBC + IL-6	19%	95%
WBC_PCT	19%	100%
PMN% + ESR	38%	79%
PMN% + CRP	38%	89%
PMN% + IL6	38%	95%
PMN% + PCT	25%	100%
ESR + CRP	75%	74%
ESR + IL-6	75%	79%
ESR + PCT	50%	90%
CRP + IL-6	69%	84%
CRP + PCT	44%	84%
IL-6 + PCT	44%	89%
WBC + PMN% + ESR	25%	79%
WBC + PMN% + CRP	25%	89%
WBC + PMN% + IL-6	19%	95%
WBC + PMN% + PCT	19%	100%
WBC + ESR + CRP	25%	74%
WBC + ESR + IL-6	19%	79%
WBC + ESR + PCT	19%	79%
WBC + CRP + IL-6	19%	84%
WBC + CRP + PCT	19%	89%
WBC + IL-6 + PCT	19%	95%
PMN% + ESR + CRP	38%	74%
PMN% + ESR + IL-6	31%	79%
PMN% + ESR + PCT	25%	79%
PMN% + CRP + IL-6	79%	84%
PMN% + CRP + PCT	25%	89%
PMN% + IL-6 + PCT	25%	95%
ESR + CRP + IL-6	69%	74%
ESR + IL-6 + PCT	50%	79%
ESR + CRP + PCT	44%	74%
CRP + IL-6 + PCT	50%	84%
WBC + PMN% + ESR + CRP	25%	74%
WBC + PMN% + ESR + IL-6	19%	79%
WBC + PMN% + ESR + PCT	19%	79%
WBC + PMN% + IL-6 + PCT	19%	95%
WBC + ESR + CRP + IL-6	19%	79%
WBC + ESR + CRP + PCT	19%	79%
WBC + CRP + IL-6 + PCT	19%	84%
PMN% + ESR + CRP + IL-6	19%	79%
PMN% + ESR + CRP + PCT	25%	79%
PMN% + CRP + IL-6 + PCT	25%	84%
ESR + CRP + IL-6 + PCT	50%	79%
WBC + PMN% + CRP + PCT	19%	89%
WBC + PMN% + CRP + IL_6	19%	84%
WBC + ESR + IL-6 + PCT	19%	79%
PMN% + ESR + IL-6 + PCT	25%	79%
WBC + PMN% + ESR + CRP + IL-6	19%	74%
WBC + PMN% + ESR + CRP + PCT	19%	74%
PMN% + ESR + CRP + IL-6 + PCT	25%	74%
WBC + ESR + CRP + IL-6 + PCT	19%	74%
WBC + PMN% + CRP + IL-6 + PCT	19%	84%
WBC + PMN% + ESR + IL-6 + PCT	19%	79%
WBC + PMN% + ESR + CRP + IL-6 + PCT	19%	74%

**Table 5:**
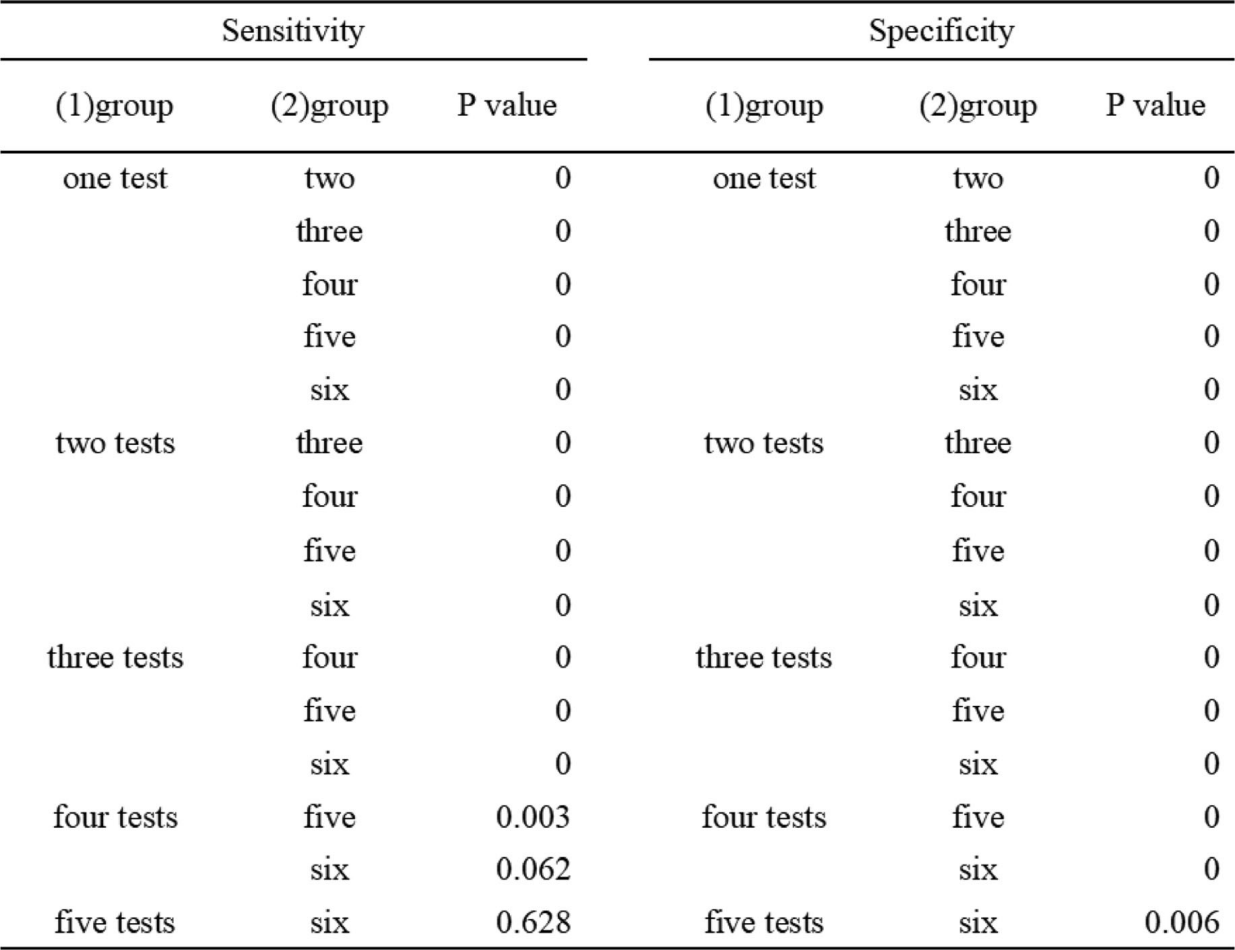
Multiple comparison of different groups

### Histopathology

**Fig. 5:**
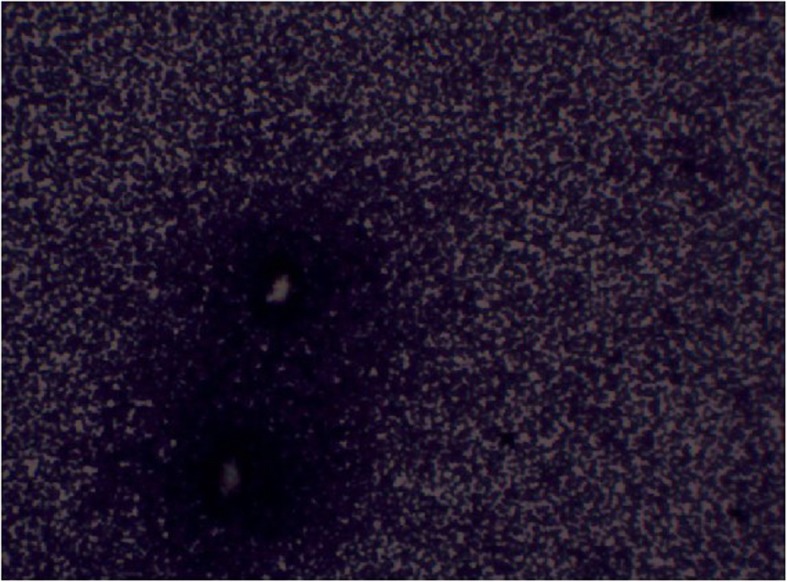
The positive control of Gram-positive bacteria

**Fig. 6:**
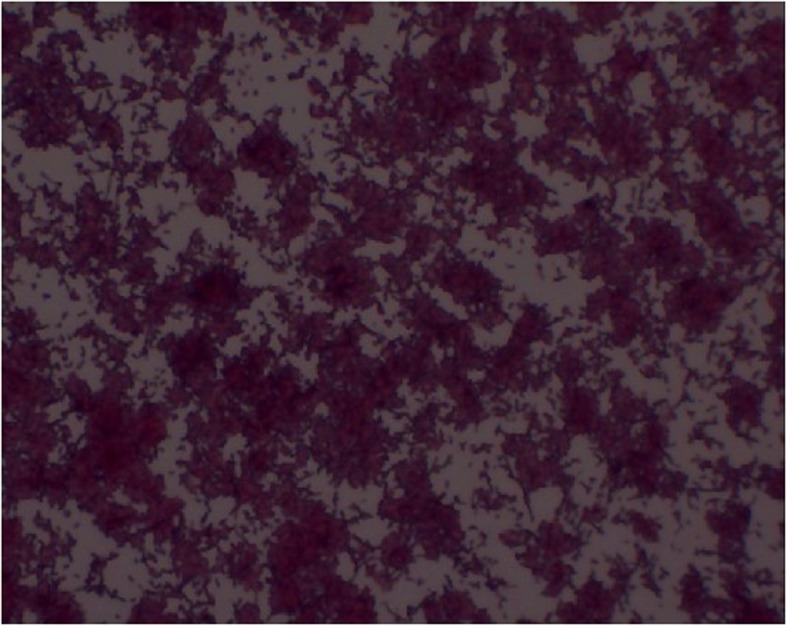
The positive control of Gram-negative bacteria

All patients had underwent revision surgery, but only 3 cases who had been suspected to PJI had carried out intraoperative pathologic tissue paraffin section, as well as 1 case in AL group. The results of all 3 PJI cases were positive to diagnosis of infection**.** There were necrosis tissue, granulation tissue, and fibrous connective tissue change to hyaline degeneration, accompanied by chronic inflammatory cells and neutrophil infiltration, and the formation of foreign body granuloma by the optical microscope. While in paraffin sections of AL case, this phenomenon did not exist, and there were a large amount of multinucleated giant cells, accompanied with the hyperplasia of fibrous connective tissue and vessel (Figs. 5 and 6).

### Aspiration

**Fig. 7:**
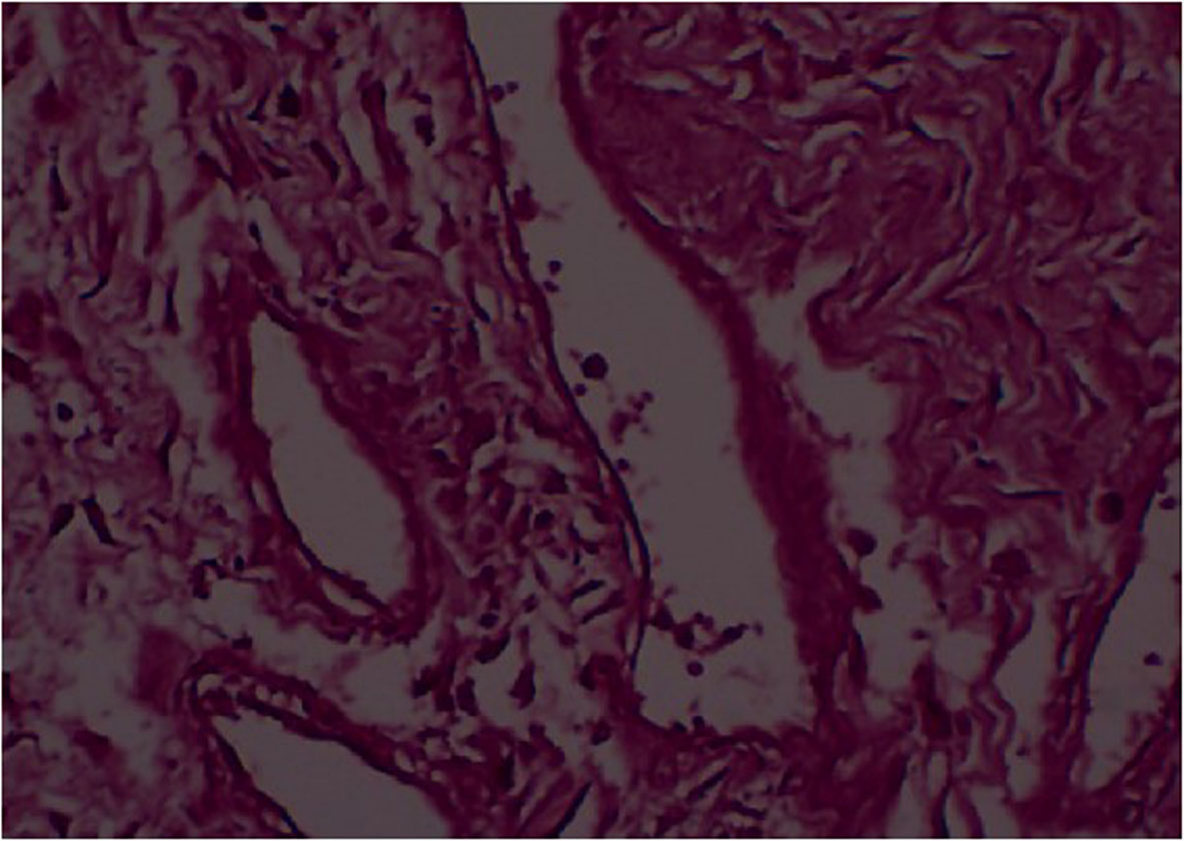
The result of Gram staining of PJI’s tissue

**Fig. 8:**
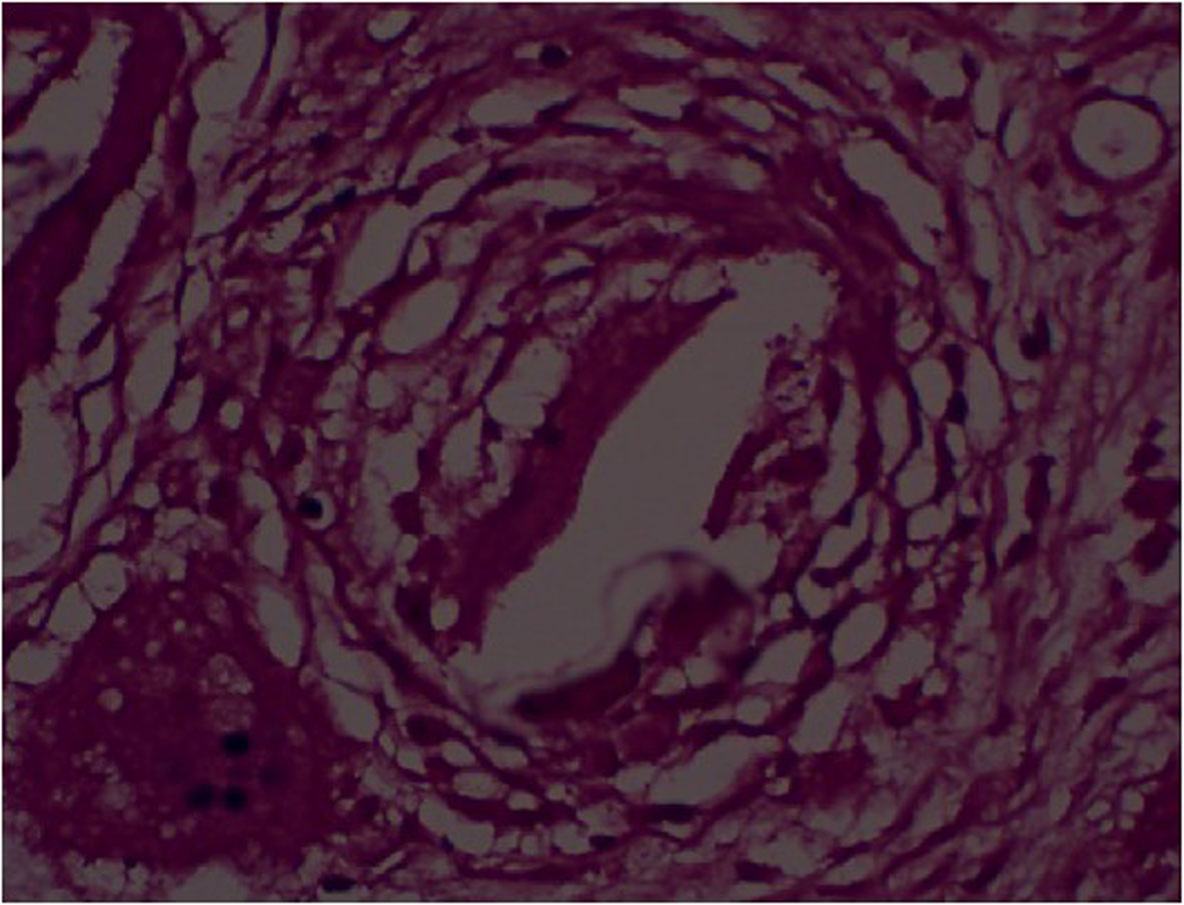
The result of Gram staining of AL’s tissue

Only 7 PJI patients had underwent aspiration, and all the joint aspiration fluid was incubated in aerobic, anaerobic, and fungal; at the same time, the synovial fluid of aspiration was measured leukocyte count and cell differentiation. According to the results of culture of aspiration fluid, we only found two positive culture results, which included *Staphylococcus epidermidis* and *Candida albicans*, and the positive rate of culture was 29%. By observing the synovial leukocyte count and neutrophil percentage (PMN%), there were 6 patients who with greater than five neutrophils per high-power field, the rate of positive was 86%, the results of positive of synovial neutrophil percentage (PMN%) were 5 cases, and the rate of positive was 71% (Figs. 7 and 8).

### Radiographic tests and ultrasonography

**Fig. 9:**
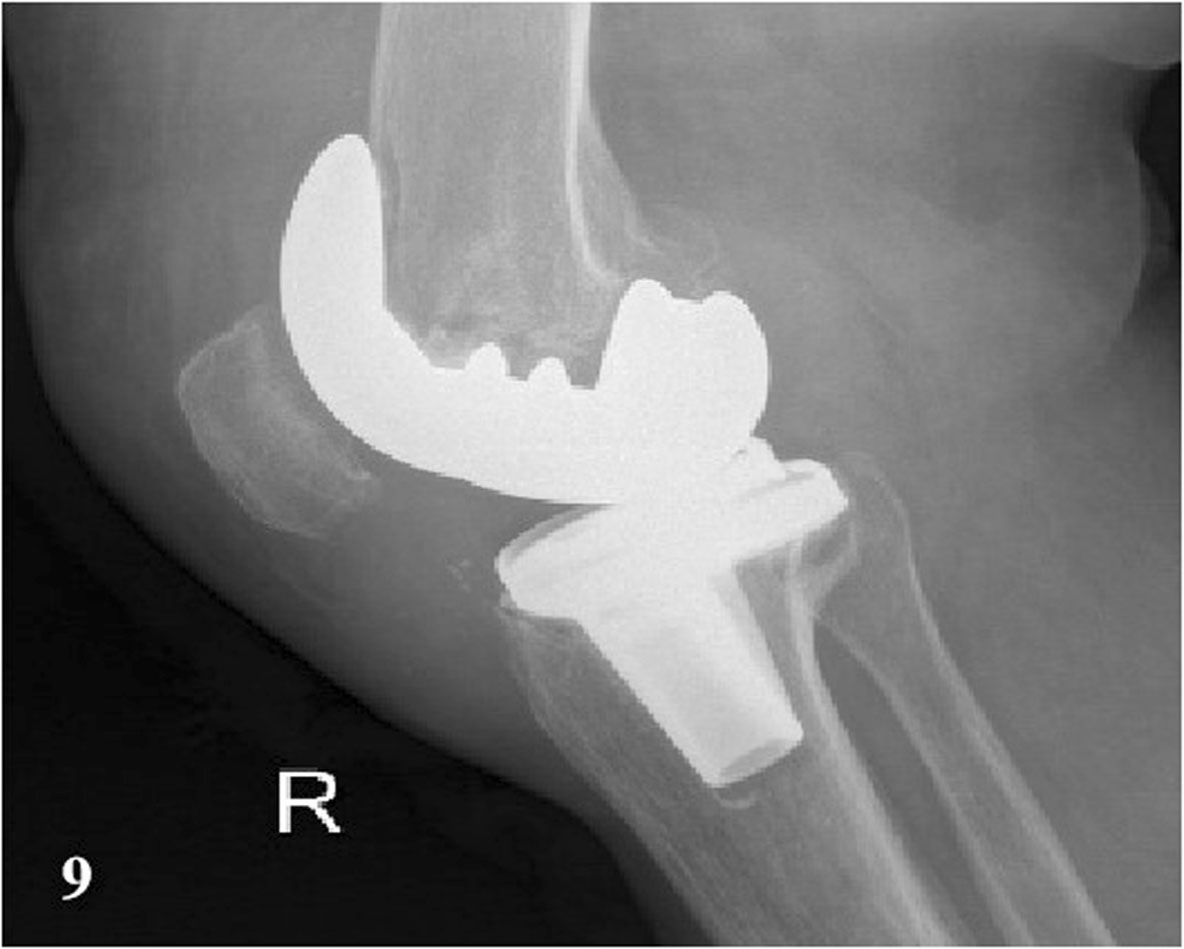
A PJI case after TKA

**Fig. 10:**
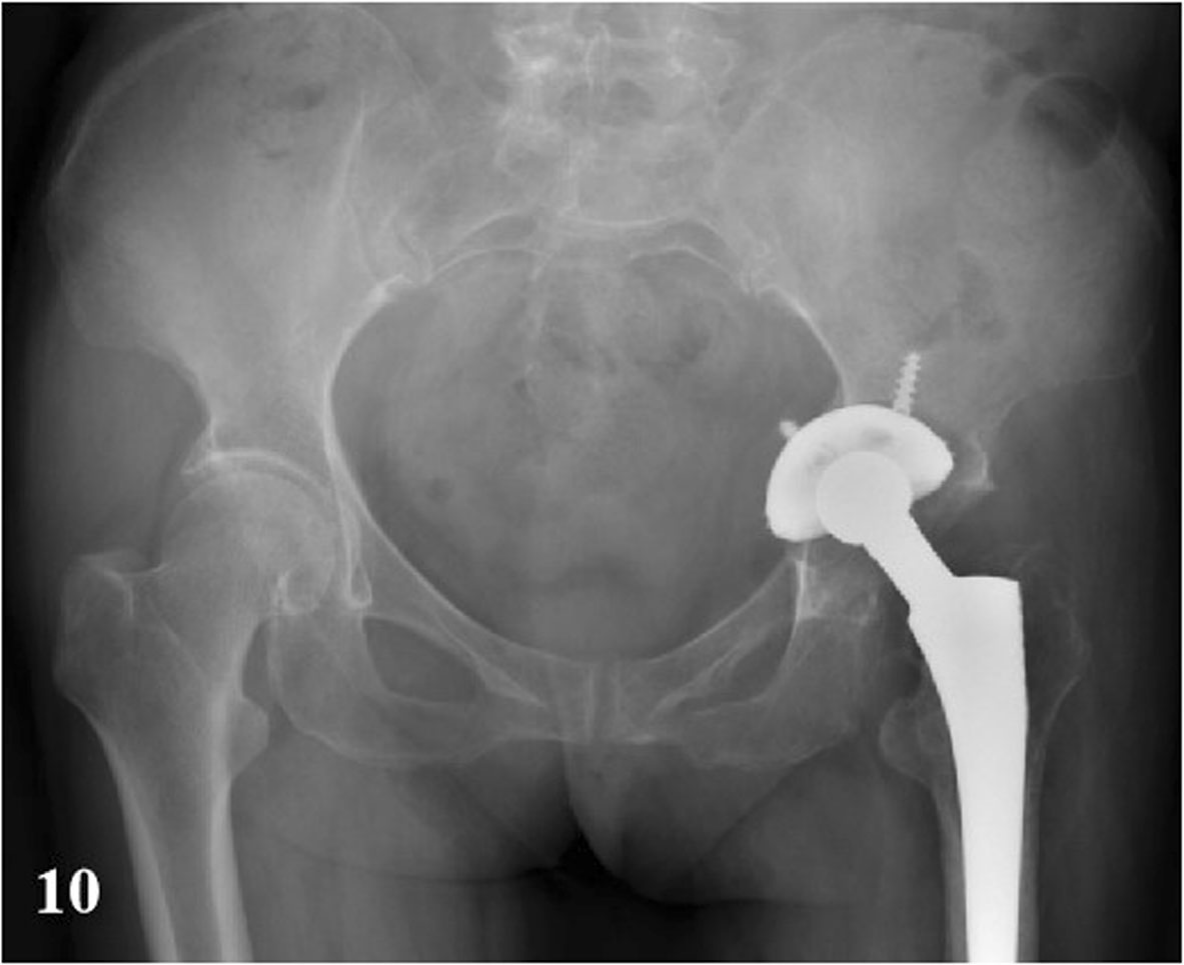
An AL case after THA

**Fig. 11:**
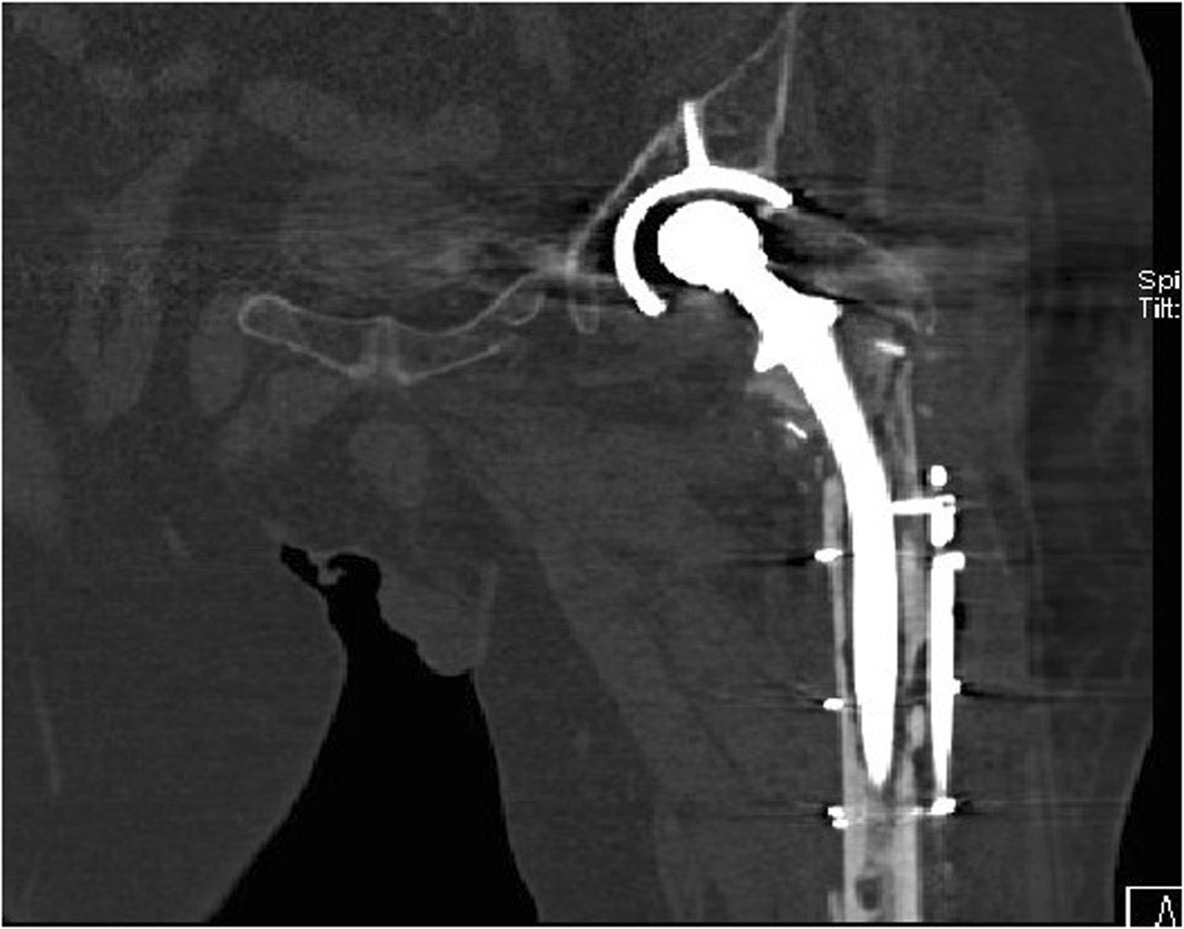
A patient with PJI after THA

**Fig. 12:**
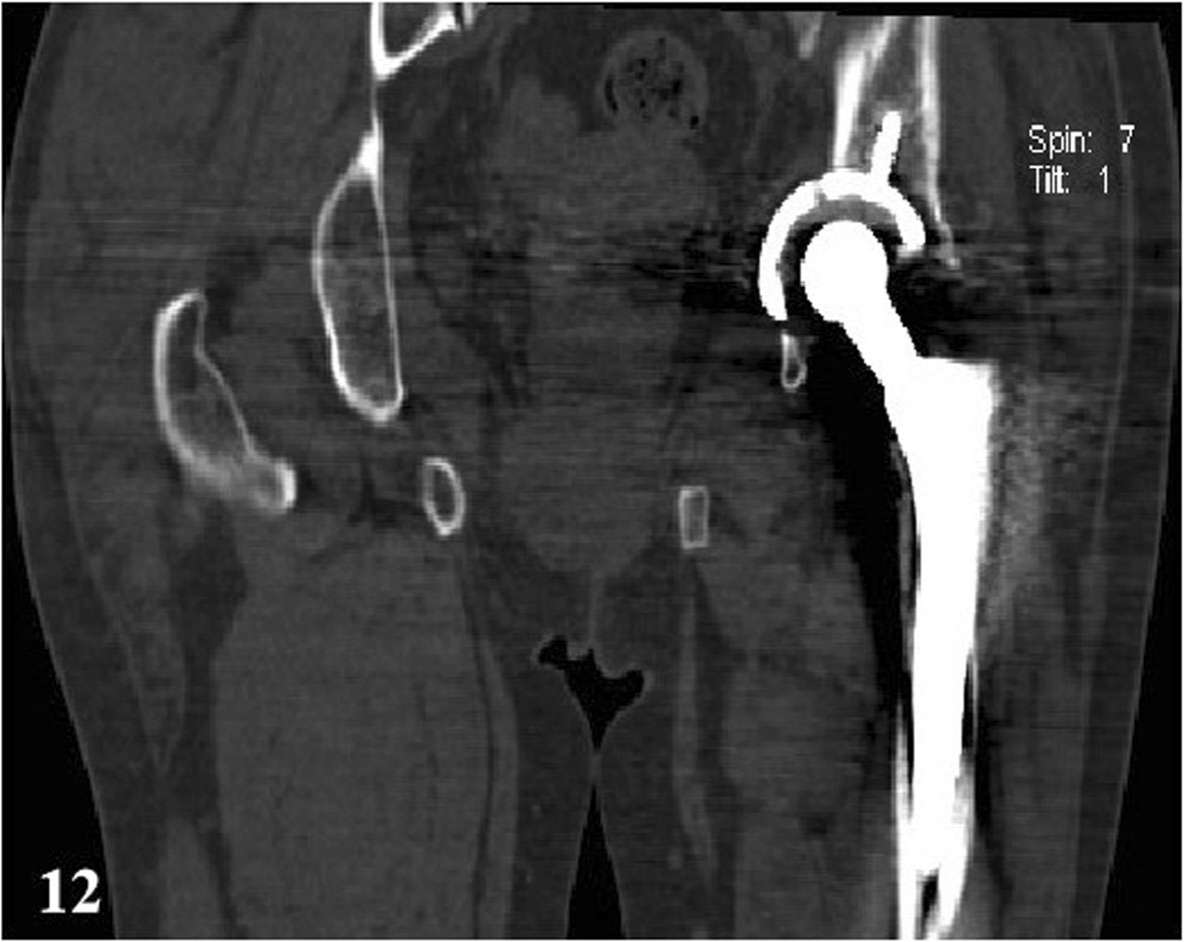
An AL case after THA

In a total of 35 patients, there were 16 PJI patients, and the others were AL cases. Before operation, X radiographs were taken on all 35 patients. In PJI group, the signs that suspected infection were found in 88% of cases (*n* = 14) (e.g., Fig. 9). The signs of loosening were apparent in 6 cases (32%) (e.g., Fig. 10). CT scans were produced of 10 PJI patients, with positive results being achieved in 9 cases (90%) (e.g., Fig. 11). CT scans were performed on 8 AL patients, with positive results being achieved in 6 cases (75%) (e.g., Fig. 12).

**Fig. 13:**
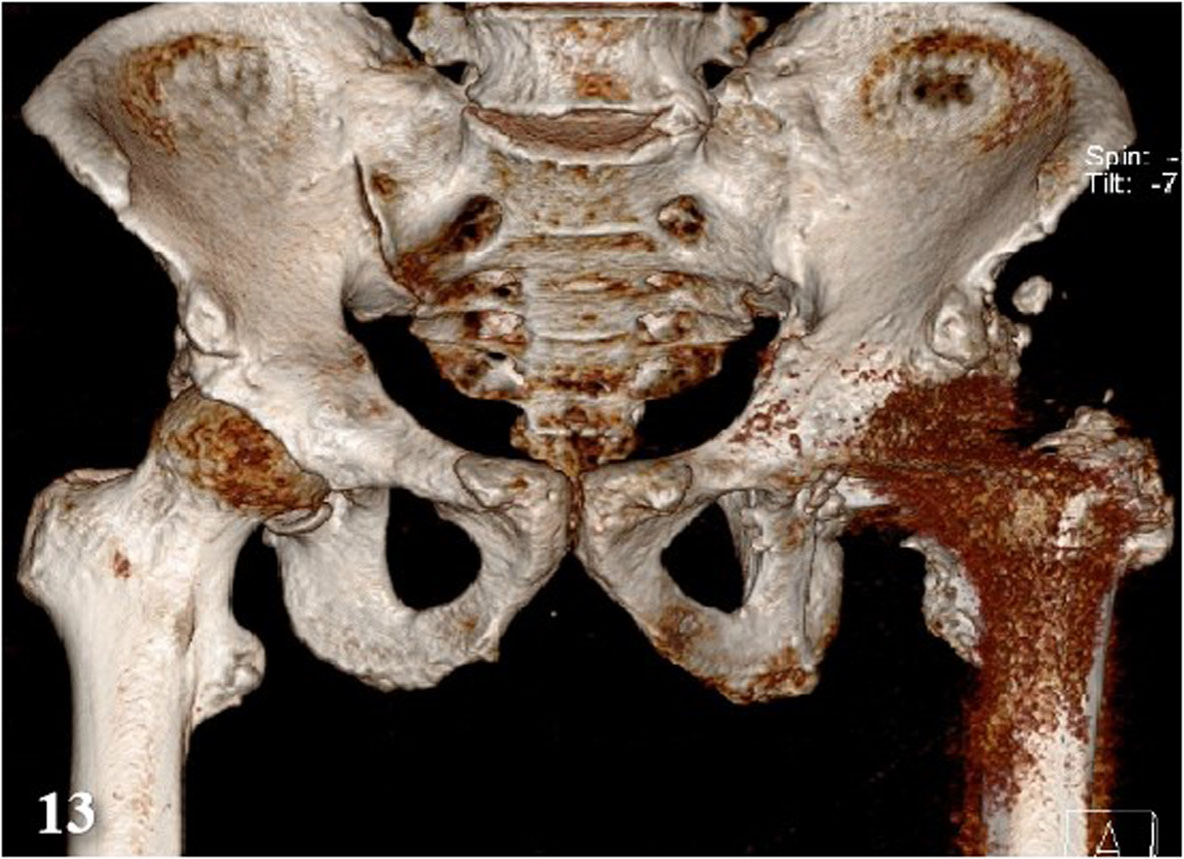
A PJI patient’s 3D CT imaging

**Fig. 14:**
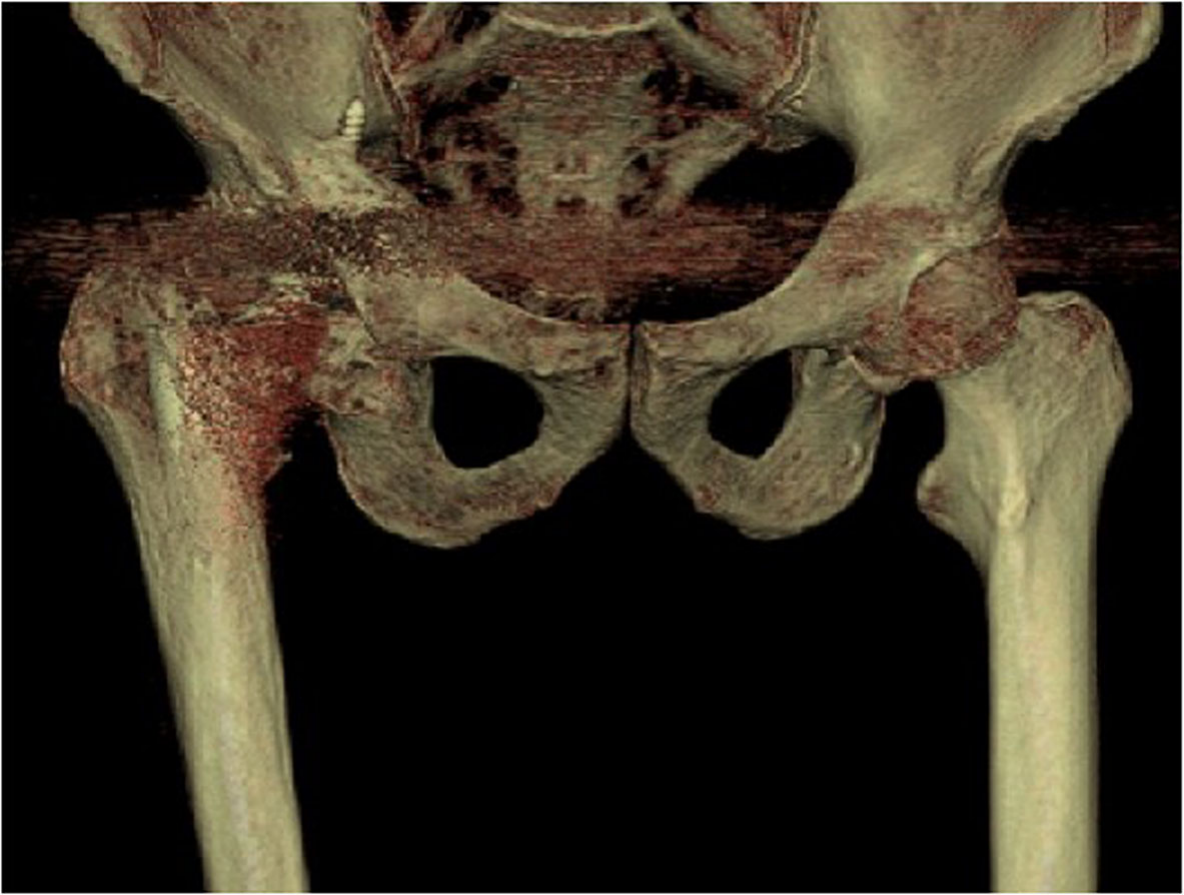
An AL patient’s 3D CT imaging

3D-CT scans were performed on 8 PJI patients, which achieved in 6 positive results (75%) (e.g., Fig. 13). And these techniques were examined in 7 AL patients as well, and there were 4 cases (57%) with positive results (e.g., Fig. 14).

**Fig. 15:**
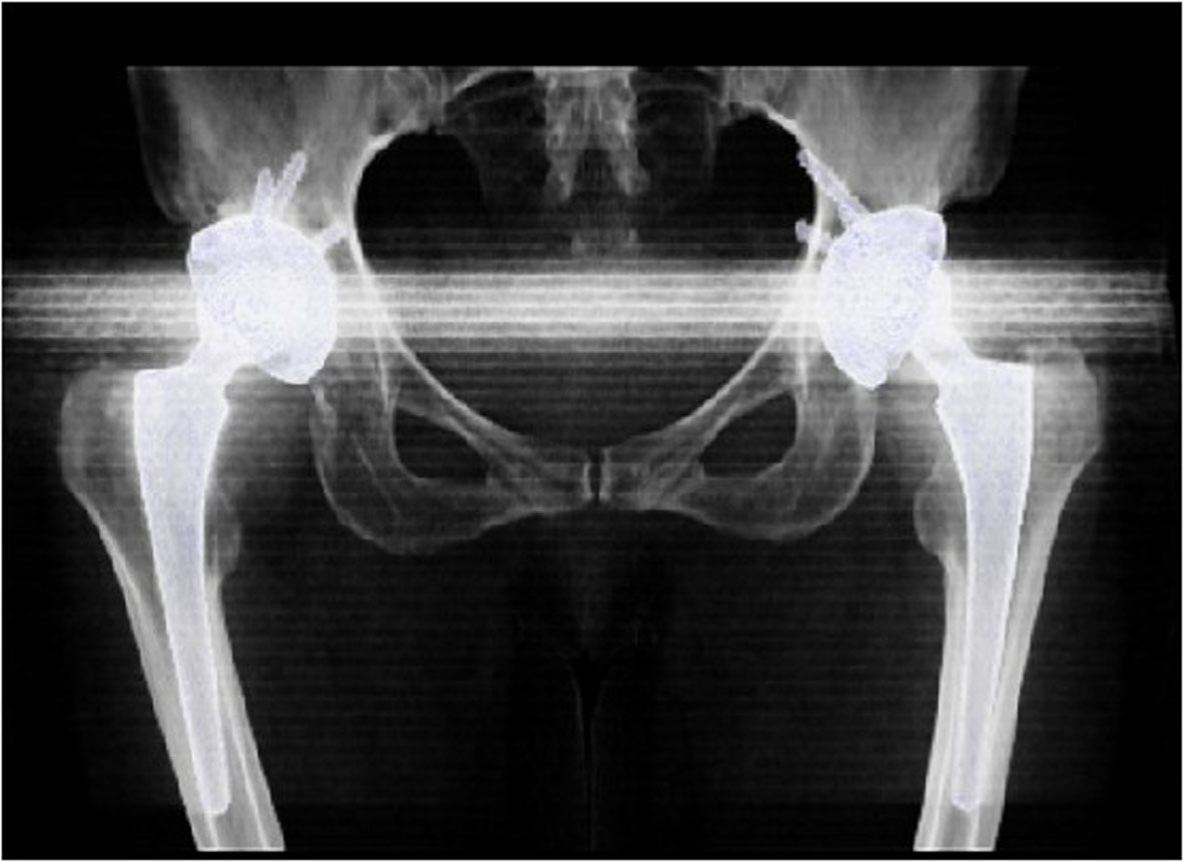
A PJI patient’s volume-rendered 3D CT imaging

**Fig. 16:**
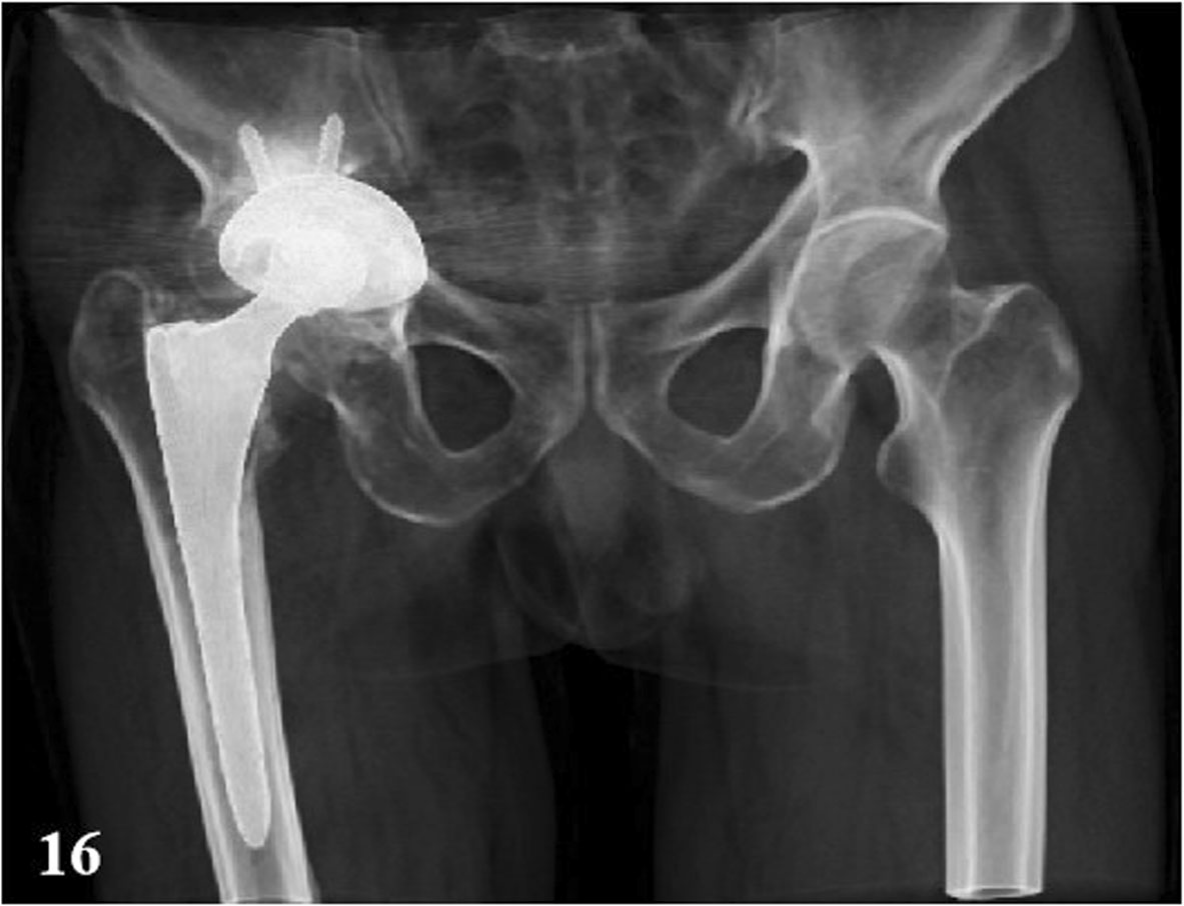
A AL patient’s volume-rendered 3D CT imaging

**Fig. 17:**
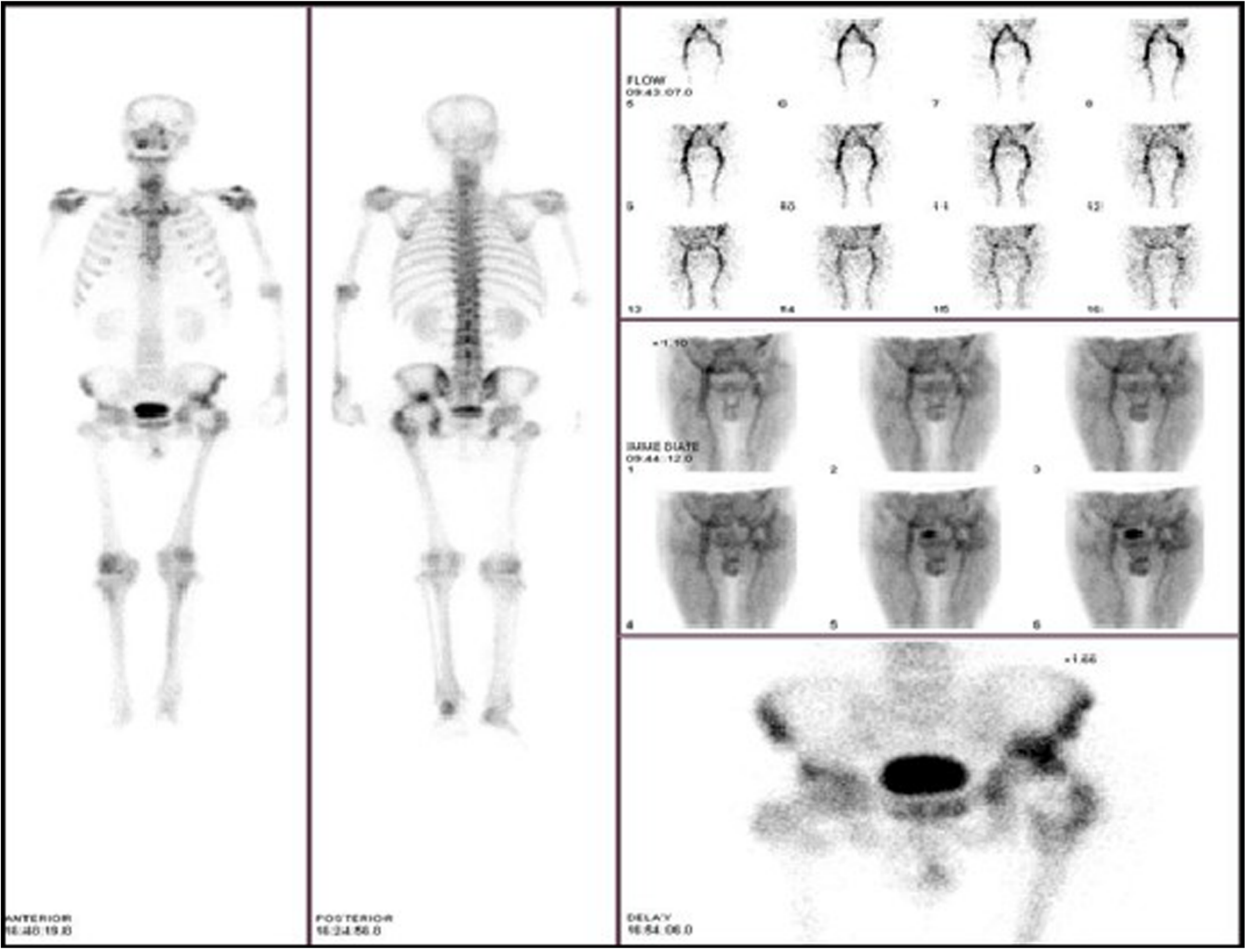
99mTc-MDP imaging of suspected PJI after THA

**Fig. 18:**
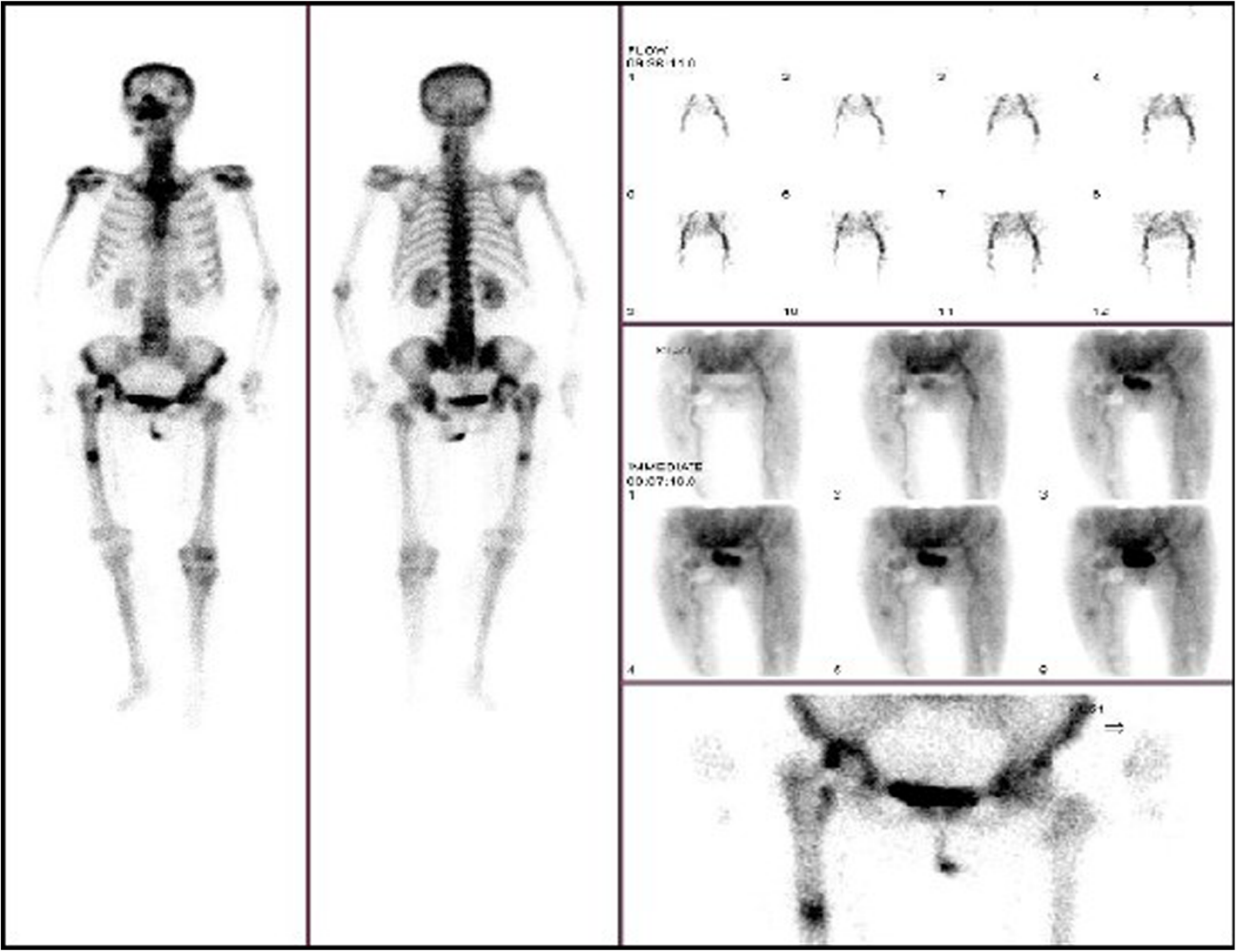
99mTc-MDP imaging of suspected AL after THA

**Fig. 19:**
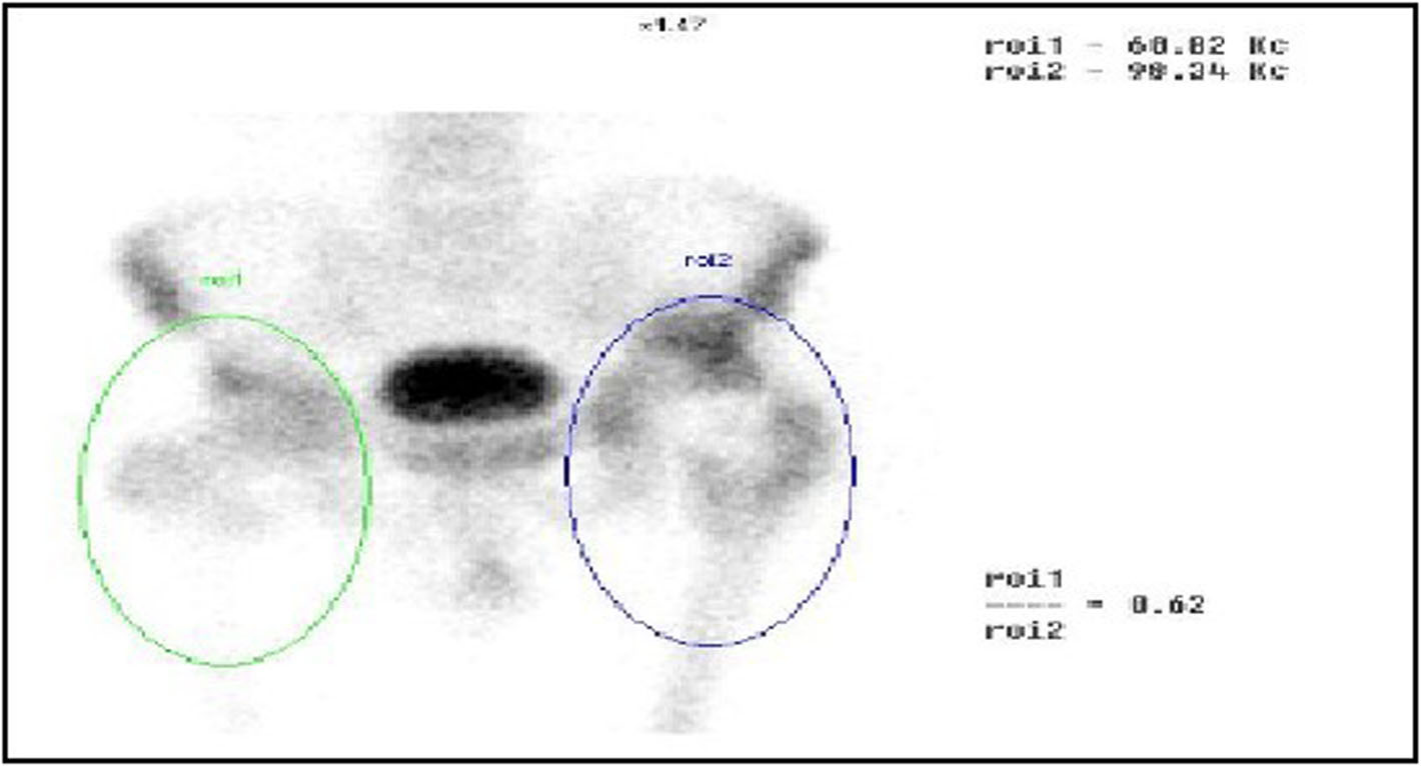
The comparison of the amount of radionuclide of bone intake of unaffected and affected sides of PJI patient. The green circle is roi1 (unaffected side), the blue one is roi2 (affected side), and the ratio of roi1/roi2 was 0.62

**Fig. 20:**
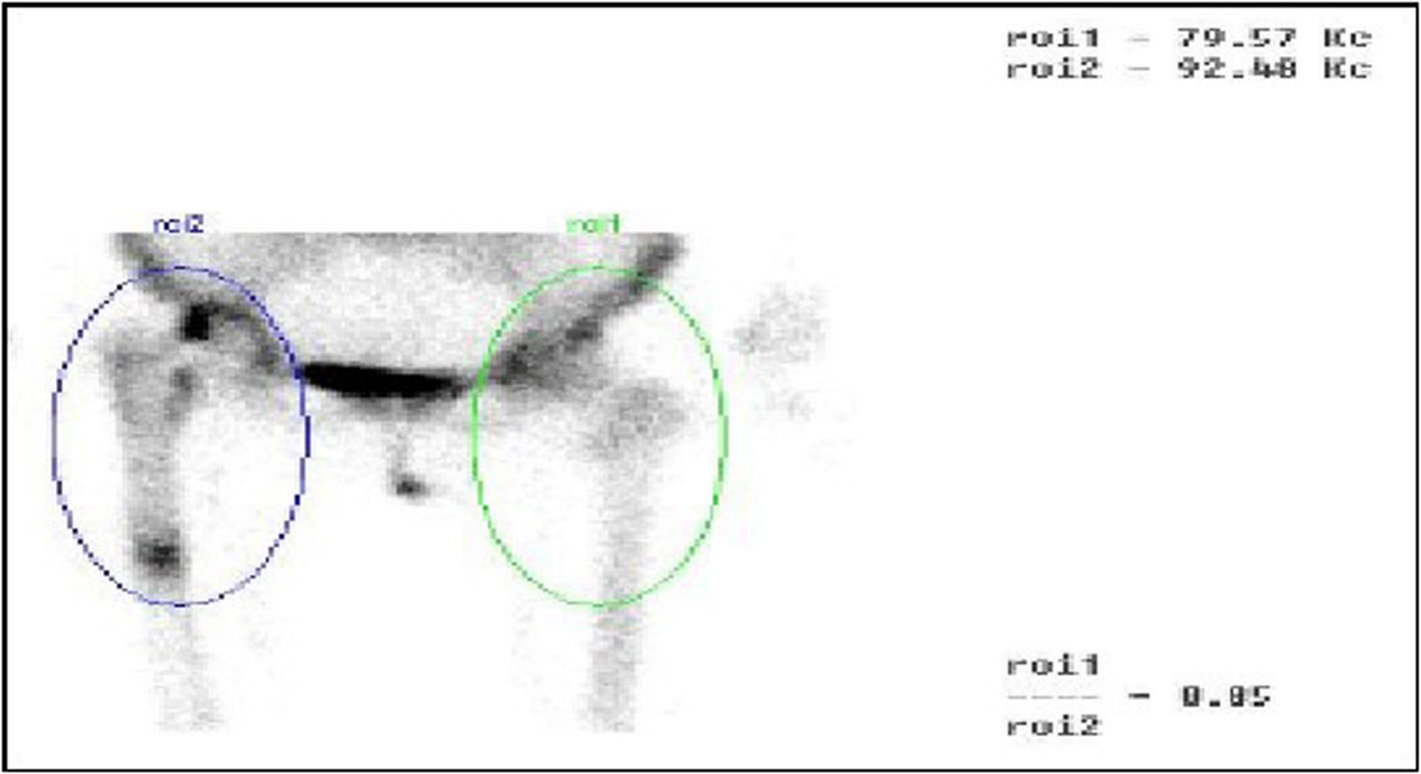
The comparison of the amount of radionuclide of bone intake of unaffected and affected sides of AL patient. The ratio of roi1/roi2 was 0.85

99mTc-MDP three-phase bone scintigraphy was used in this study, 99mTc-MDP was carried out on 3 suspected PJI patients and 1 suspected AL patients (Figs. 15 and 16), and all these 4 patients (100%) achieved in positive results (e.g., Figs. 17 and 18). By comparison in the amount of radionuclide of bone intake of unaffected and affected sides of PJI and AL patients, we also found that the amount of radionuclide of bone intake of PJI patient is higher (e.g., Figs. 19 and 20).

**Fig. 21:**
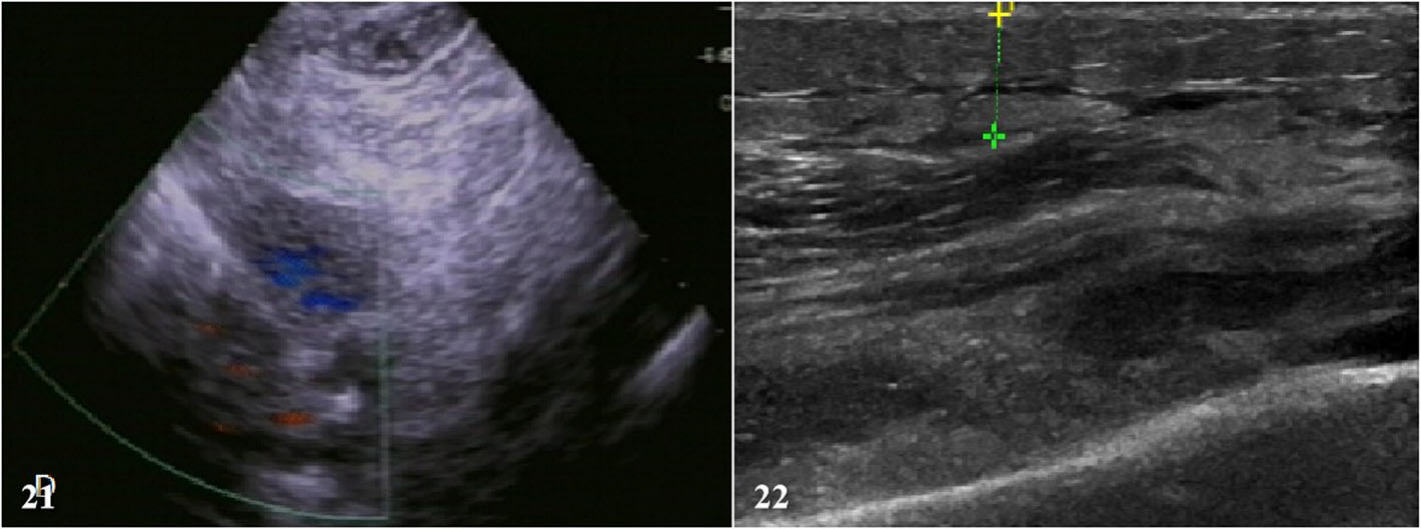
Ultrasound scan on the right knee joint which was suspected to PJI

**Fig. 22:**
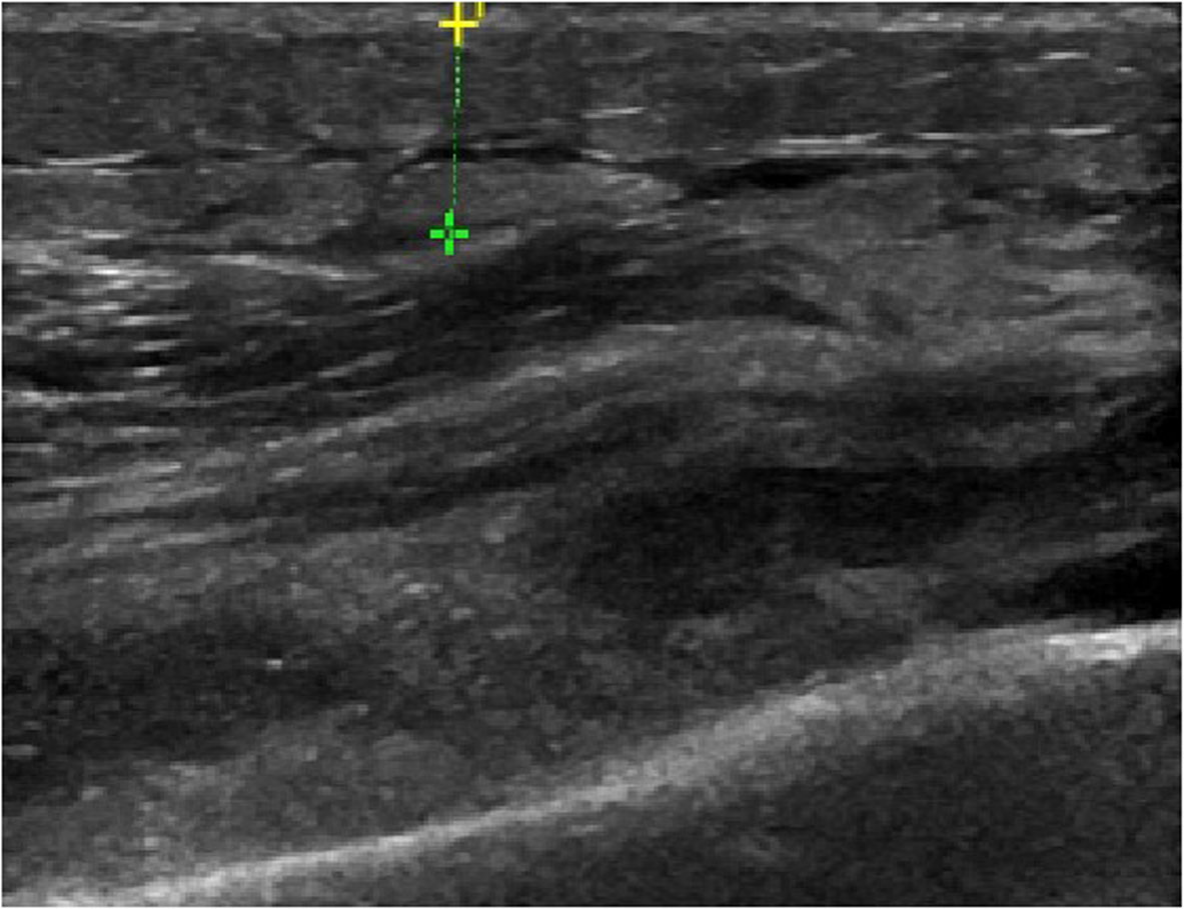
Ultrasound scan on the left knee joint which was suspected to AL case after TKA

Ultrasound scan was performed for evaluating the conditions of periprosthetic collections after THA and TKA. There were 5 patients who were suspected to PJI that had underwent the examination of ultrasound scans, and 4 (80%) of them achieved in positive results (e.g., Fig. 21). And as well as ultrasound scans were performed on 2 patients who with AL, but none of them had positive results (e.g., Fig. 22).

## Discussion

The diagnosis of PJI still remains a challenge, though for which many preoperative and intraoperative tests have been employed. Unfortunately, none of current tests has perfect sensitivity and specificity to diagnosis PJI. Our study is the first retrospective multiple factor study to explore the diagnostic value of different tests in the southwest of China.

For laboratory tests, we evaluated the SN, SP, PPV, and NPV of WBC, PMN%, CRP, ESR, IL-6, and PCT, respectively, analyzed the combinations of all different six tests, and calculated the accuracy to diagnosis PJI of different tests. Strikingly, we found that the highest sensitivity of tests was ESR and CRP, the value was 81%, the highest value of specificity and PPV were all 100%, and all these were WBC, whereas the highest value of NPV was LI-6 (86%). According to the ROC curves and AUC of six kinds of tests, we could know that CRP yields to a high-quality diagnostic value to PJI, and ESR, IL-6, PCT, WBC, and PMN% yield to a medium-quality diagnostic value. This result was resembled with several previous studies [9, 10]. Some previous studies had mentioned that ESR and CRP were representative serologic values for diagnosing infection [11, 12]. However, the values were not absolute indications of infection because CRP could be influenced by many factors [5]. With the up-and-coming tests and the combinations of different tests, which had showed different sensitivities and specificities to diagnosis of PJI, we found that the combination of PMN%, CRP, and IL-6 had the highest sensitivity (79%), while the highest value of specificity was 100%; this result was the same as to the combinations of WBC + PMN%, WBC + PCT, PMN% + PCT, and WBC + PMN% + PCT, and there were no significant difference in sensitivity when we combined any five kinds of tests or more to together to diagnosis PJI, and this phenomenon did not exist in specificity.

According to the results of pathologic tissue paraffin section, only 3 cases who had been suspected to PJI had carried out intraoperative pathologic tissue paraffin section, and as well as 1 case in AL group. Besding on the definition of PJI which was published by MSIS [13], the positive value of pathologic tissue paraffin section was 100% (3 cases), and in all PJI cases could found chronic inflammatory cells and neutrophil infiltration by the optical microscope, as well as the negative value was 100%. For the diagnostic methods of histopathology, excepting to paraffin tissue sections, there is intraoperative frozen section as well, and the American Academy of Orthopaedic Surgeons also strongly recommended that the frozen section is useful for ruling in PJI when such a diagnosis cannot be made preoperatively [14]. In recent years, frozen section, owing to its inexpensive cost, simple procedure, and timely report, has become a promising and important tool for detecting PJI, but in our study, we did not examined the FS. Some recent reports have shown that this diagnostic method has a high accuracy compared with preoperative tests [15]. Meanwhile, some studied also made some comparisons between the histopathology of frozen and paraffin section, and several reports indicated that analysis of frozen sections agrees with that of paraffin sections [16], whereas the others showed that there were apparent difference in the sensitivity and specificity between frozen sections and paraffin sections or other tests [7, 17]. Regarding to the discrepancy between the findings from frozen and paraffin sections, which might due to differences in the quality of the sections in this study, we also adopt the technology of Gram staining, the result of Gram staining is optimistic, so we strongly recommended that the technology of Gram staining as the diagnostic method to diagnosis PJI.

One sign of PJI is joint effusions and fluid collections surrounding the hardware. Ultrasonography had dynamic capabilities over other cross-sectional imaging modalities, with the dynamic capabilities, that make it possible to evaluate for periprosthetic collections as well as evaluate the regional relationships between the soft-tissue structures and implantable hardware [8]. In our study, Power Doppler was used to detect the presence of these soft-tissue fluid collections, if there was a relatively thick hypoechoic band around the prosthesis on routine gray-scale imaging. Five patients who were suspected to PJI had underwent the examination of ultrasound scans, and 4 (80%) of them achieved in positive results. And as well as ultrasound scans were performed on 2 patients who with AL, but none of them had positive results.

In summary, all the above tests which we have analyzed showed the different sensitivities and specificities and contribute different diagnostic value to distinguish PJI and AL. According to the viewpoints that we summarized, we attempt to find a best way to diagnosis PJI, but without getting satisfactory results, we hope there will be an ideal method to diagnosis in the future day, and with the characterized of inexpensive cost, simple procedure, and timely report, maybe it is a kind of serologic test, molecular method, or radiographic test.

There are several notable limitations to this study. The major limitations of this study are that the sample size of patients with PJI and AJ is low, so the sensitivity and specificity of laboratory tests may be lack of specificity. With the incidence of PJI is low, so we still cannot recruit more patients, the way that can solve this problem is to prolong the time of study or unite multicenter in the southwest of China to complete this study.

## Data Availability

The datasets generated during the current study are public at the email 215069125@qq.com.

## Abbreviations

PJI: Periprosthetic Joint Infections
TKA: Total knee arthroplasty
THA: Total hip arthroplasty
MSIS: Musculoskeletal Infection Society
AL: Aseptic Loosening
WBC: White blood cell
PMN: Polymorphonuclear
CRP: C-reactive protein
ESR: Erythrocyte sedimentation rate
PCT: Procalcitonin
MRI: Magnetic resonance imaging
CT: Computed tomography
AL: Aseptic loosening
IL-6: Interleukin-6
SN: Sensitivity
SP: Specificity
PPV: Positive predictive value
NPV: Negative predictive value
99mTc: Technetium-99 m
MDP: Methylene diphosphonate
ROC: Receiver operating characteristic curve
AUC: Area under the curve
